# Supplementary material for: The highly developed symbiotic system between the solar-powered nudibranch Pteraeolidia semperi and Symbiodiniacean algae
Source: iScience. 2023 Nov 15;26(12):108464. doi: 10.1016/j.isci.2023.108464 (PMC10730344; doi:10.1016/j.isci.2023.108464)
Supplement: Document S1. Figures S1–S3 and Data S1–S3 [file mmc1.pdf]

**iScience, Volume *26***

## Supplemental information

# The highly developed symbiotic system between the solar-powered nudibranch *Pteraeolidia semperi* and Symbiodiniacean algae

**Hideaki Mizobata, Kenji Tomita, Ryo Yonezawa, Kentaro Hayashi, Shigeharu Kinoshita, Kazutoshi Yoshitake, and Shuichi Asakawa**

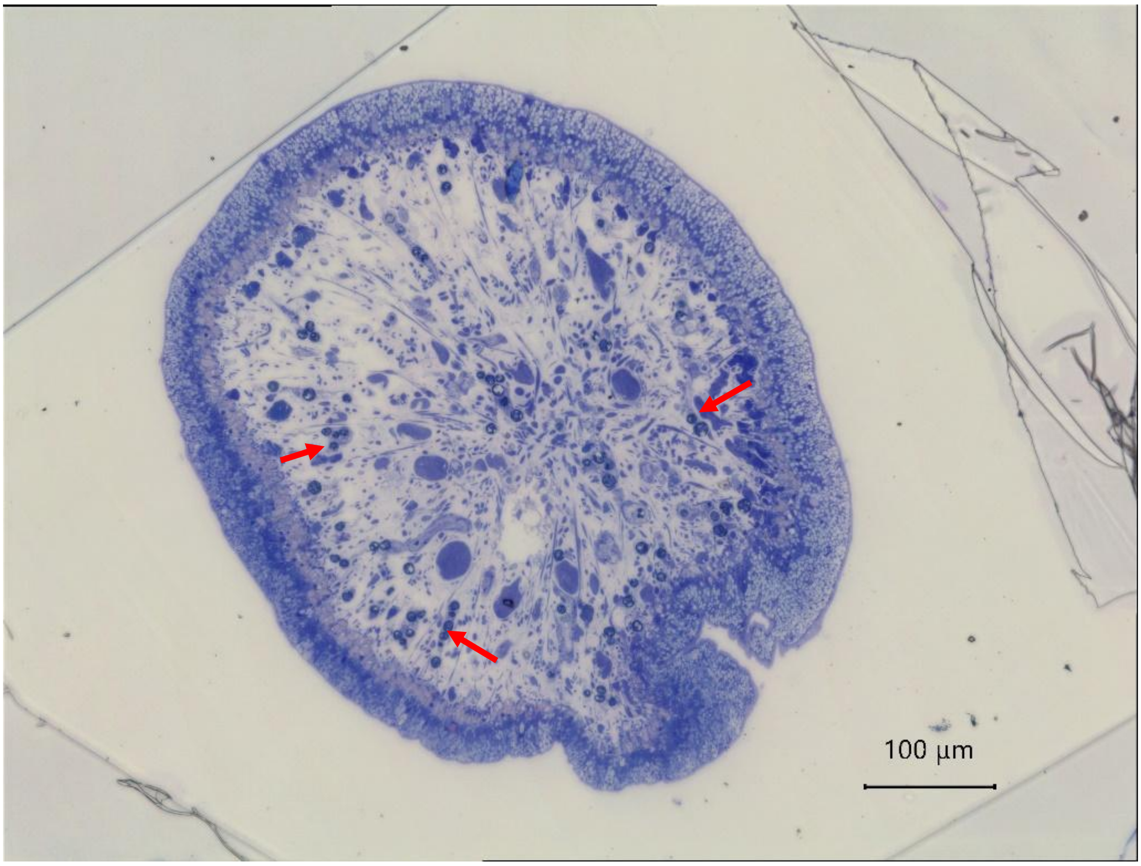


**Figure S1. Cross-section of a tentacle from an unbleached individual subjected to 15 days of starvation, related to Figure 2h. Some of the algal cells are indicated by red arrows.**

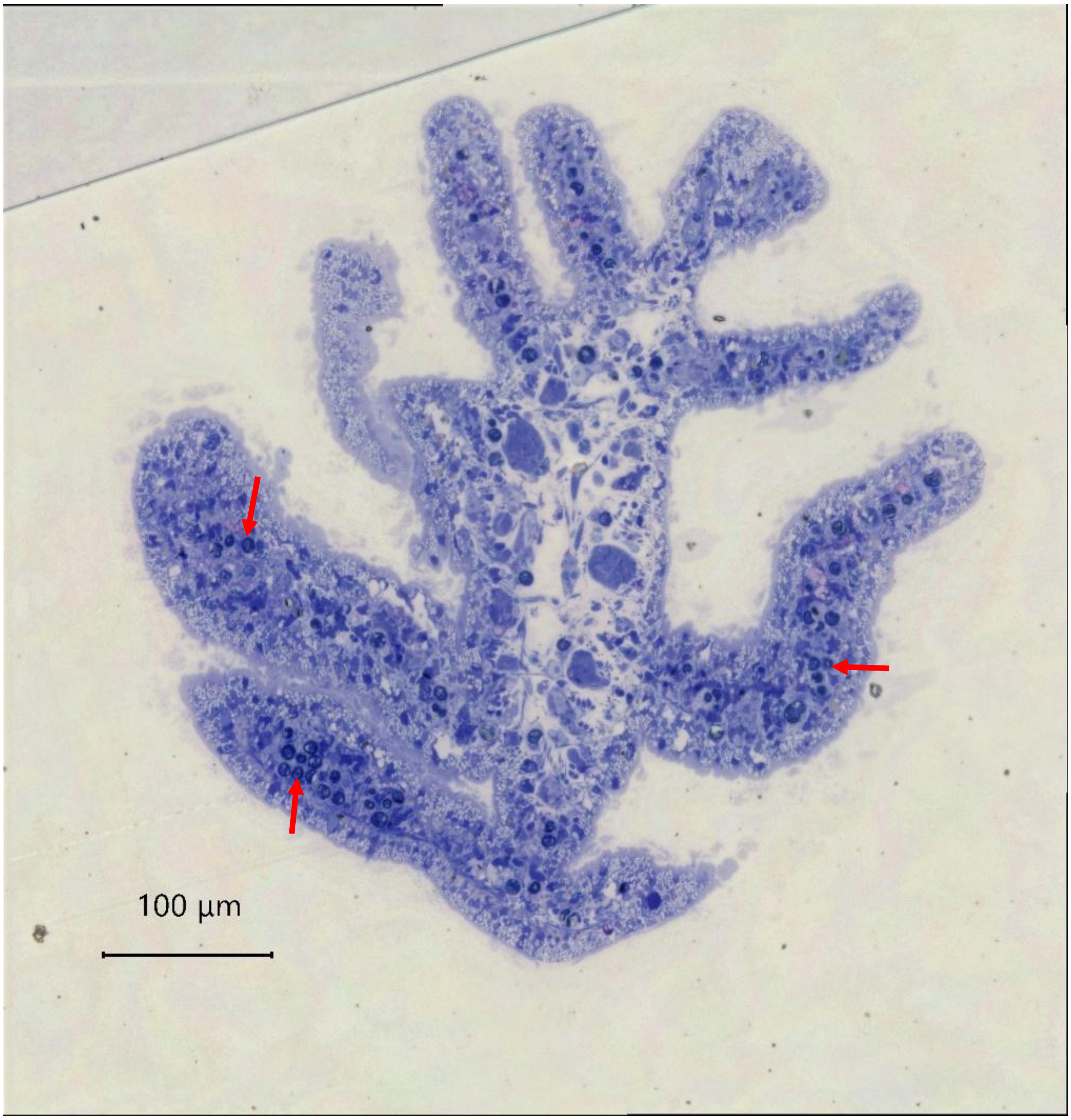


**Figure S2. Cross-section of a rhinophore from an unbleached individual subjected to 15 days of starvation, related to Figure 2h. Some of the algal cells are indicated by red arrows.**

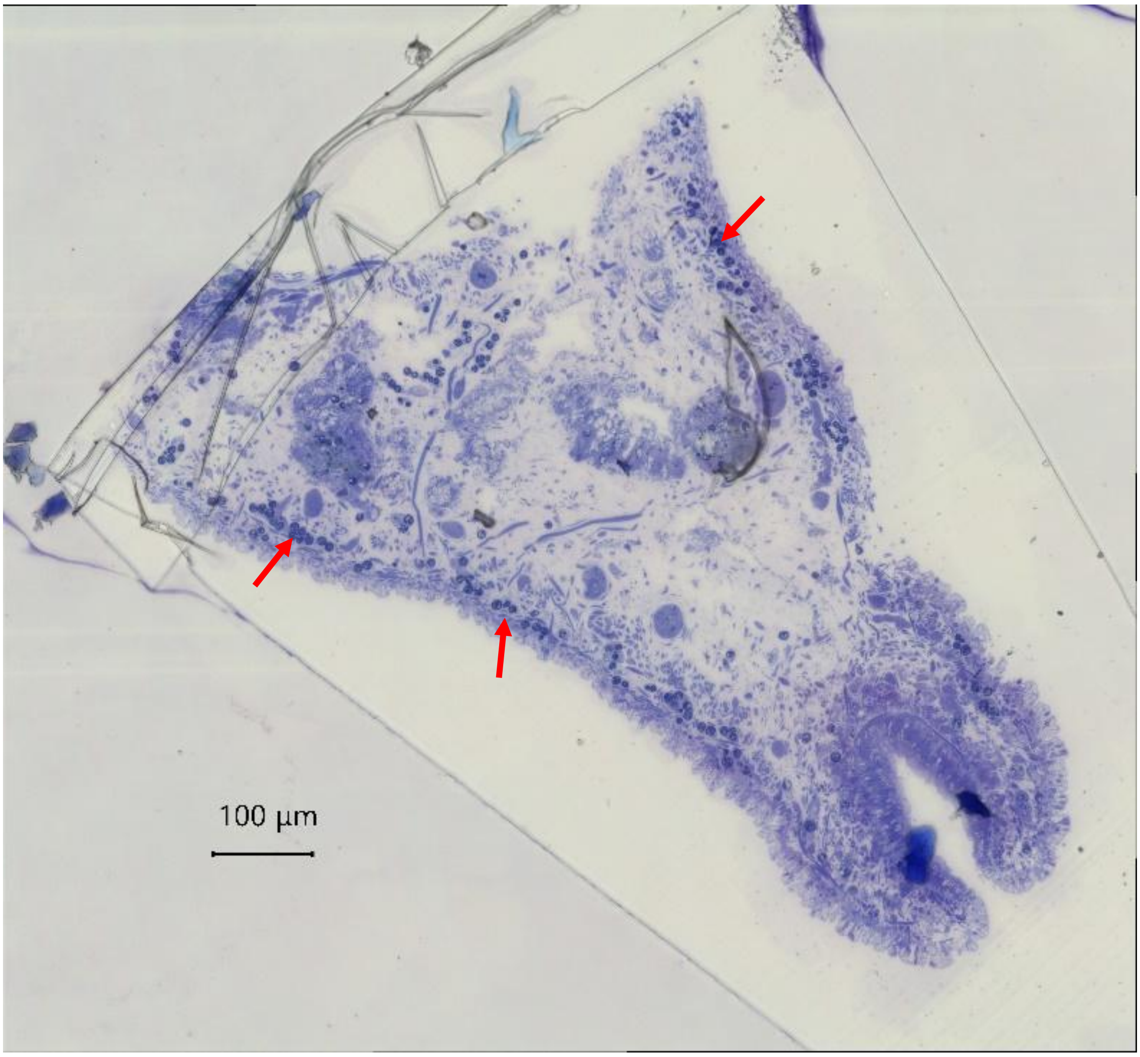


**Figure S3. Cross-section of a body from an unbleached individual subjected to 15 days of starvation, related to Figure 2h. Some of the algal cells are indicated by red arrows.**

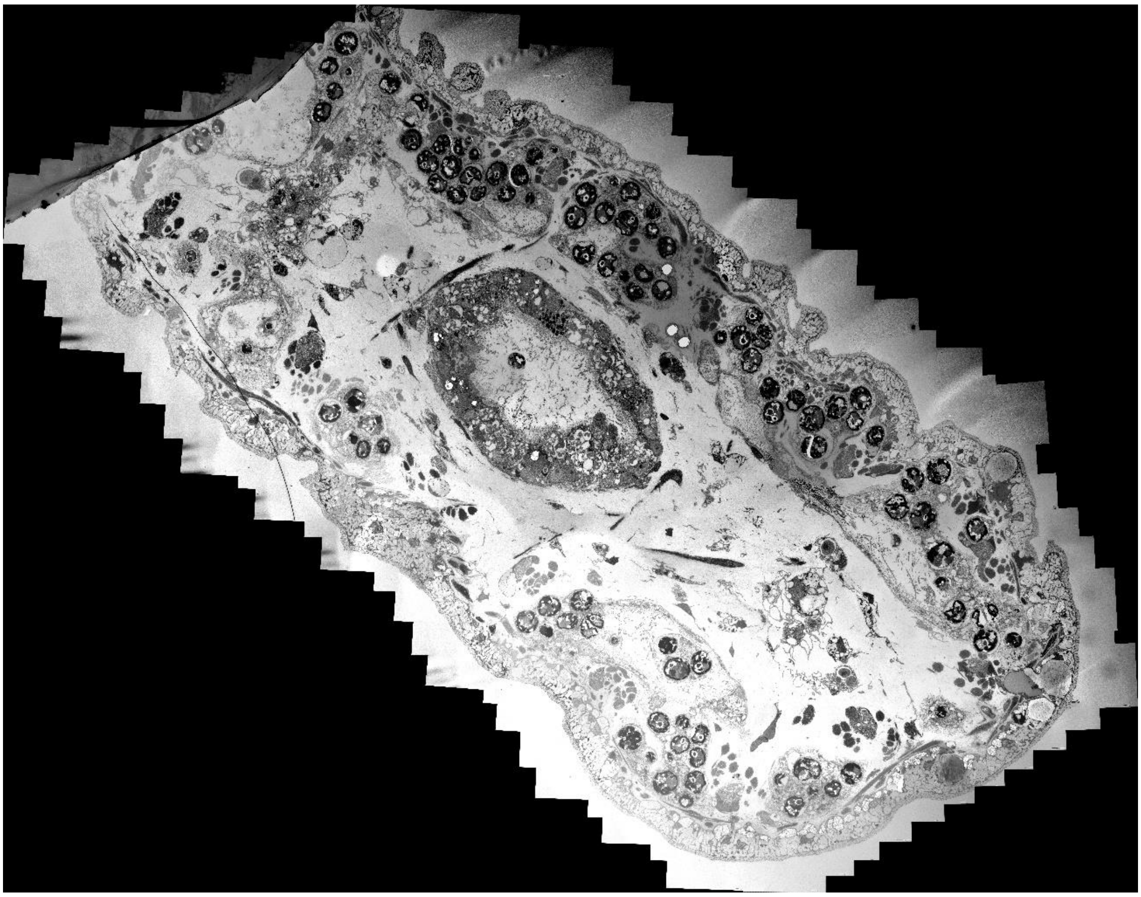

**Data S1. Combined TEM images of a cerata cross-section from an unbleached individual subjected to 15 days of starvation, related to Figure 3b. The embedded image is of low resolution; however, the original is of ultra-high resolution, allowing for detailed observation of cell microstructures upon magnification. The original high-resolution image can be accessed via Figshare (https://doi.org/10.6084/m9.figshare.22734740).**

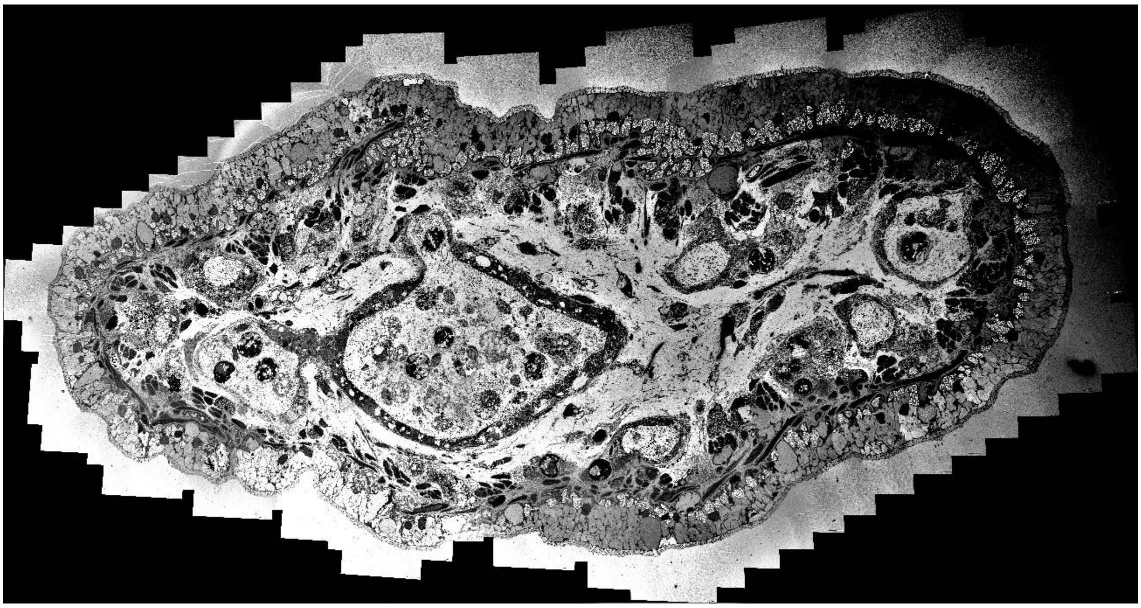

**Data S2. Combined TEM images of a cerata cross-section from a bleached individual subjected to 50 days of starvation, related to Figure 3c,d. The embedded image is of low resolution; however, the original is of ultra-high resolution, allowing for detailed observation of cell microstructures upon magnification. The original high-resolution image can be accessed via Figshare (https://doi.org/10.6084/m9.figshare.22734740).**

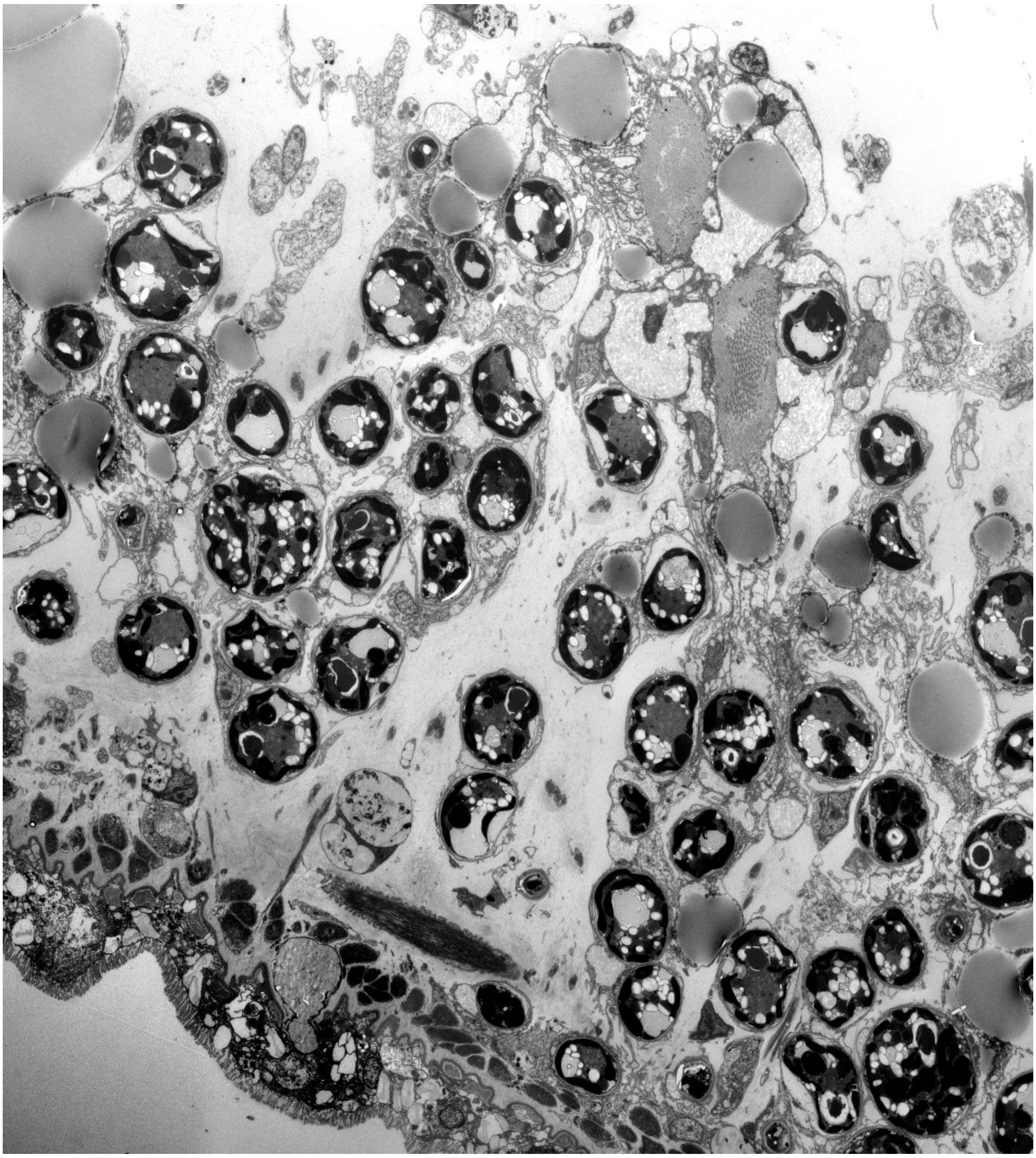

**Data S3. Combined TEM images of a cerata longitudinal section from a fresh individual (unbleached), related to Figure 3a. The embedded image is of low resolution; however, the original is of ultra-high resolution, allowing for detailed observation of cell microstructures upon magnification. The original high-resolution image can be accessed via Figshare ([https://doi.org/10.6084/m9.figshare.22734740](https://doi.org/10.6084/m9.figshare.22734740)).**
